# Susceptibility-Weighted Imaging of the Anatomic Variation of Thalamostriate Vein and Its Tributaries

**DOI:** 10.1371/journal.pone.0141513

**Published:** 2015-10-27

**Authors:** Xiao-fen Zhang, Jian-ce Li, Xin-dong Wen, Chuan-gen Ren, Ming Cai, Cheng-chun Chen

**Affiliations:** 1 Department of Human Anatomy, Wenzhou Medical University, Wenzhou, Zhejiang, China; 2 Department of Radiology, the 1^st^ Affiliated Hospital of Wenzhou Medical University, Wenzhou, Zhejiang, China; 3 Department of Neurosurgery, the 2^nd^ Affiliated Hospital of Wenzhou Medical University, Wenzhou, Zhejiang, China; Shenzhen institutes of advanced technology, CHINA

## Abstract

**Background and Purpose:**

Thalamostriate vein (TSV) is an important tributary of the internal cerebral vein, which mainly drains the basal ganglia and deep medulla. The purpose of this study was to explore the anatomic variation and quality of TSV and its smaller tributaries using susceptibility-weighted imaging (SWI).

**Methods:**

We acquired SWI images in 40 volunteers on a 3.0T MR system using an 8-channel high-resolution phased array coil. The frequencies of the TSV and its tributaries were evaluated. We classified TSV into types I (forming a venous angle) and II (forming a false venous angle). We classified anterior caudate vein (ACV)into types 1 (1 trunk) and 2 (2 trunks) as well as into types A (joiningTSV), B (joining anterior septal vein), and C (joining the angle of both veins).

**Results:**

The TSV drains the areas of caudate nucleus, internal capsule,lentiform nucleus, external capsule, claustrum, extreme capsule and the white matter of the frontoparietal lobes,except thalamus. The frequencies of the TSV, ACV and transverse caudate vein (ACV) were 92.5%, 87.5% and 63.8%, respectively. We found TSV types I and II in 79.7%, and 20.3% with significantly different constitution ratios (*P*< 0.05). The most common types of ACV were type 1 (90.0%) and type A (64.3%).

**Conclusion:**

The complex three-dimensional (3D) venous architecture of TSV and its small tributaries manifests great variation, with significant and practical implications for neurosurgery.

## Introduction

Susceptibility-weighted imaging (SWI) is a relatively new MR imaging technique based on variation in blood oxygenation between venous blood andsurrounding cerebral parenchyma [1]. The differences in magnetic susceptibility between oxygenated and deoxygenated haemoglobin are delineated by SWI in terms of large (diameter approximately 1mm) and small (diameter less than 1 mm) veins in the brain using a long TE, 3D gradient-echo MR sequence [2]. Digital subtraction angiography (DSA) remains the gold standard for measurement of vessel dimensions *invivo*, but is an invasive technique showing unilateral veins [3]. The most widely used and relatively appropriate technique is magnetic resonance venography (MRV) which is limited in its ability to visualize small vessels [4]. SWI is significantly superior to MRV [4] with regard to smaller venous structures and DSA [5], without the need for intravenous contrast agent. In recent decades, several studies have used the technique to investigate deep cerebral veins [5, 6], cerebellar veins [7]and spinal veins [8, 9].

Thalamostriate vein (TSV) is the largest tributary of internal cerebral vein, which mainly drains the areas of basal nuclei and frontoparietal white matter. In neurosurgery of the third ventricle, TSV or theforamen of Monro is frequently used as a ventricular landmark [10, 11]. We often occluded TSV for a wider exposure to the third ventricle [12]. However, Mohamed *et al*. [13] suggest that deliberate occlusion of TSV should be performed only when absolutely necessary to avoid the risk of infarcts of the basal nuclei. Therefore, a thorough understanding of the anatomic variation of TSV and its tributaries is imperative.

To our knowledge, studies investigating the anatomy of TSV and its tributaries are limited. The purpose of this study was to explore the normal anatomy of TSV and its anatomic variations and small tributaries using SWI *invivo*.

## Materials and Methods

### Volunteer Selection

A total of 40 healthy adult volunteers (22 Females and 18 males; age range, 20–35; mean age, 26) were included. The absence of cerebral and other intracranial diseaseswas confirmed in all volunteers. All participants signed informed consent and were informed of the potential side effects of 3.0T MRI, including vertigo, nausea, and claustrophobia. The study design was approved by the Ethics Committee of Wenzhou Medical University.

### MRI

All healthy volunteers were examined on a 3T MR system (Royal Philips Electronics, Amsterdam, The Netherlands)using an 8-channelhigh-resolution phased array coil. The following sequences were performed: (1)T1-weighted imaging(T1WI) and fluid-attenuated inversion recovery(FLAIR) sequence(repetition time [TR]1900 ms, echo time [TE] 20 ms, flip angle 90°, matrix 256 ×141, section thickness 6 mm, gap between sections 1 mm and field of view [FOV] 230 mm); (2)T2-weighted imaging (T2WI) and turbo spin-echo (TSE) sequence(TR 2100 ms, TE 80 ms, flip angle 90°, matrix 352 × 285, section thickness 6 mm, gap between sections 1 mm and FOV 230 mm); (3)T2 FLAIR sequence (TR 6000 ms, TE 123 ms, flip angle 90°, matrix 268 × 143, section thickness 6 mm, gap between sections 1 mm and FOV 230 mm); (4)diffusion-weighted imaging (DWI)(TR2600 ms, TE 89 ms, flip angle 90°, matrix 128×128, section thickness 6 mm, gap between sections 1 mm and FOV 230 mm); (5)magnetic resonance venography (MRV) used venographic 3D principal component analysis including sensitivity(VEN-3D-PCA-SENSE) (TR 17 ms, TE 6 ms, flip angle 10°, matrix 192 × 116, NEX 1,section thickness 1 mm and FOV 230 mm); and (6)SWI(VEN-BOLD) (TR 21 ms, TE 32 ms, flip angle 10°, matrix 316 × 362, section thickness 1 mm, gap between sections -0.5 mm and FOV 220 mm).

### Image Processing

Images were processed using the Extended MR WorkSpace release 2.6.3.4 workstation (Philips). The SWI images were reformatted using minimum intensity projections (mIPs) technique with contiguous 20 mm-thick sections and -19 mm section gap in the transverse, sagittal and coronal planes. The final thickness of the sections was 1 mm.

### Evaluation

The bilateral TSV and small tributaries were evaluated bythree experienced neuroradiologistsin the transverse, sagittal and coronal planes of SWI. We classified the anatomic variation of TSV and anterior caudate veins (ACV) as follows.

### Evaluation of TSV

We classified TSV into 2 types called Type I and Type II based on a previous study [14].

**Fig 1:**
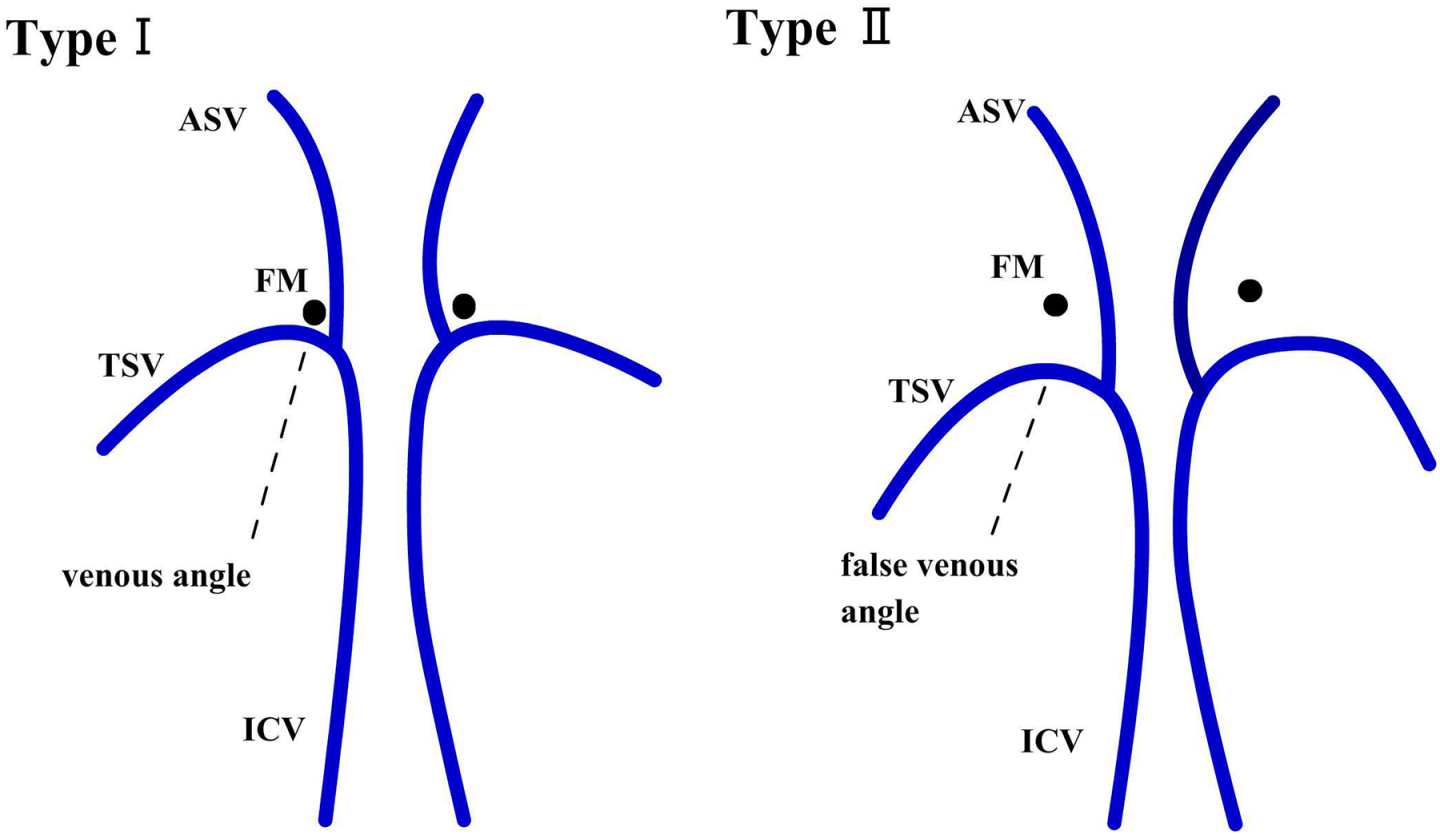
Diagrammatic representation of TSV types I and II containing venous angle and false venous angle, respectively. (ICV, internal cerebral vein; ASV, anterior septal vein; TSV, thalamostriate vein; FM, Foramen of Monro).

Type I: The U-shaped junction of TSV and the internal cerebral vein was adjacent to the posterior margin of the foramen of Monro forming the venous angle (Fig 1).

Type II: The U-shaped junction of TSV and the internal cerebral vein extended beyond the posterior margin of the foramen of Monro forming a false venous angle (Fig 1).

### Evaluation of ACV

**Fig 2:**
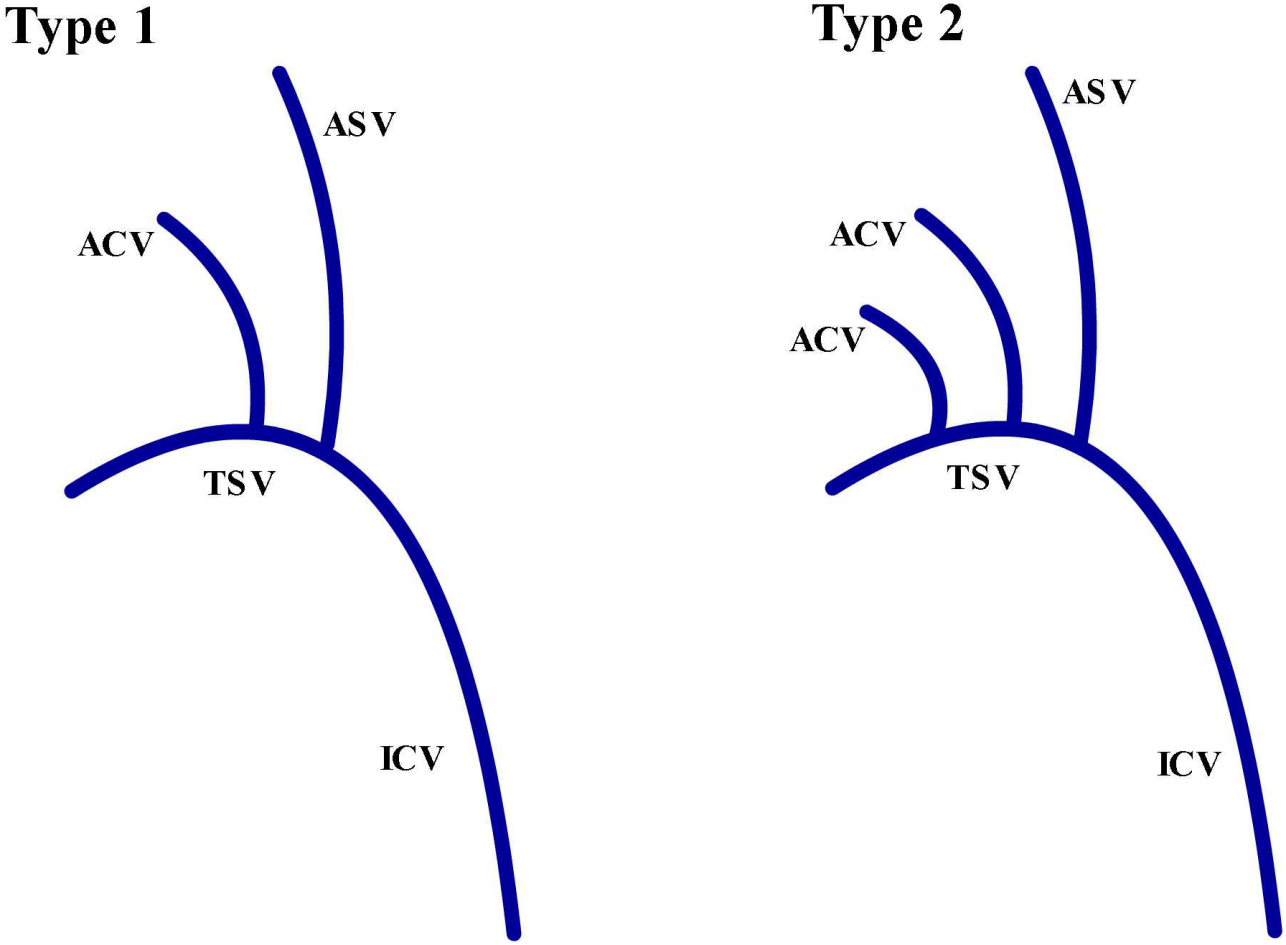
Diagram representingtwo different types of ACV. Type 1 contains 1 trunk; Type 2 contains 2 trunks. (ICV,internal cerebral vein; ASV,anterior septal vein; TSV, thalamostriate vein; ACV, anterior caudate vein).

**Fig 3:**
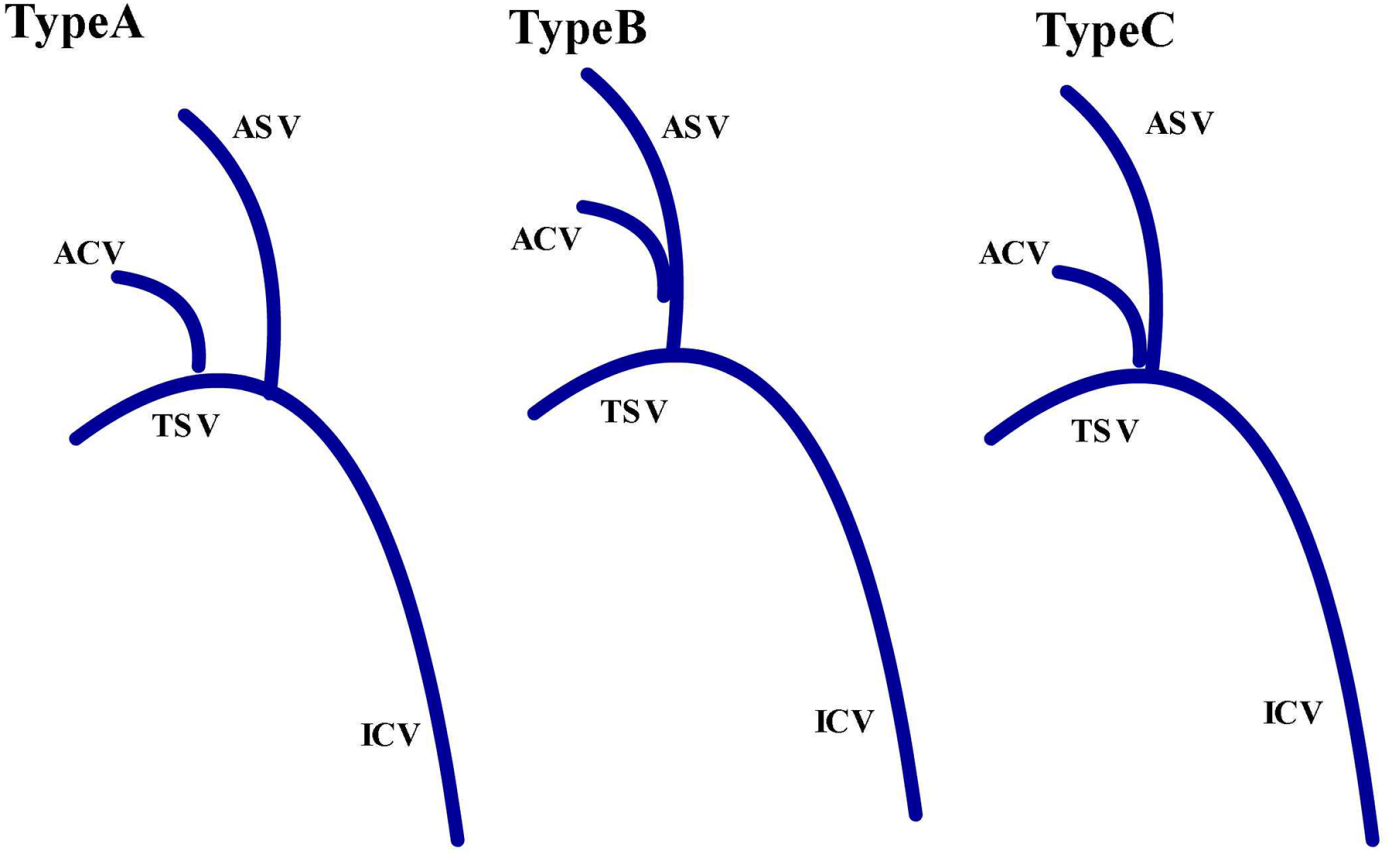
Diagram representingthree different types of ACV. Type A, joining the TSV; Type B, joining the ASV; Type C, joining the angle of TSV and ASV (ICV,internal cerebral vein; ASV,anterior septal vein; TSV, thalamostriate vein; ACV, anterior caudate vein).

We classified ACV into 2 types according to the number of trunks (Type1 = 1, Type2 = 2) (Fig 2) and 3 types (Types A, B, and C) based on the terminal joining position (Fig 3). Type A involvesACVs joining TSV. In type B, the ACVs join the anterior septal vein (ASV). The type C ACVs are attached to the angle of TSV and ASV.

### Statistical Analysis

The differences in anatomic variation of both hemispheres were assessed using the χ^2^ test, with a value of *P*< 0.05 considered statistically significant. Data were analyzed using SPSS version 16.0 (IBMSPSS, Chicago, IL, USA).

## Results

**Fig 4:**
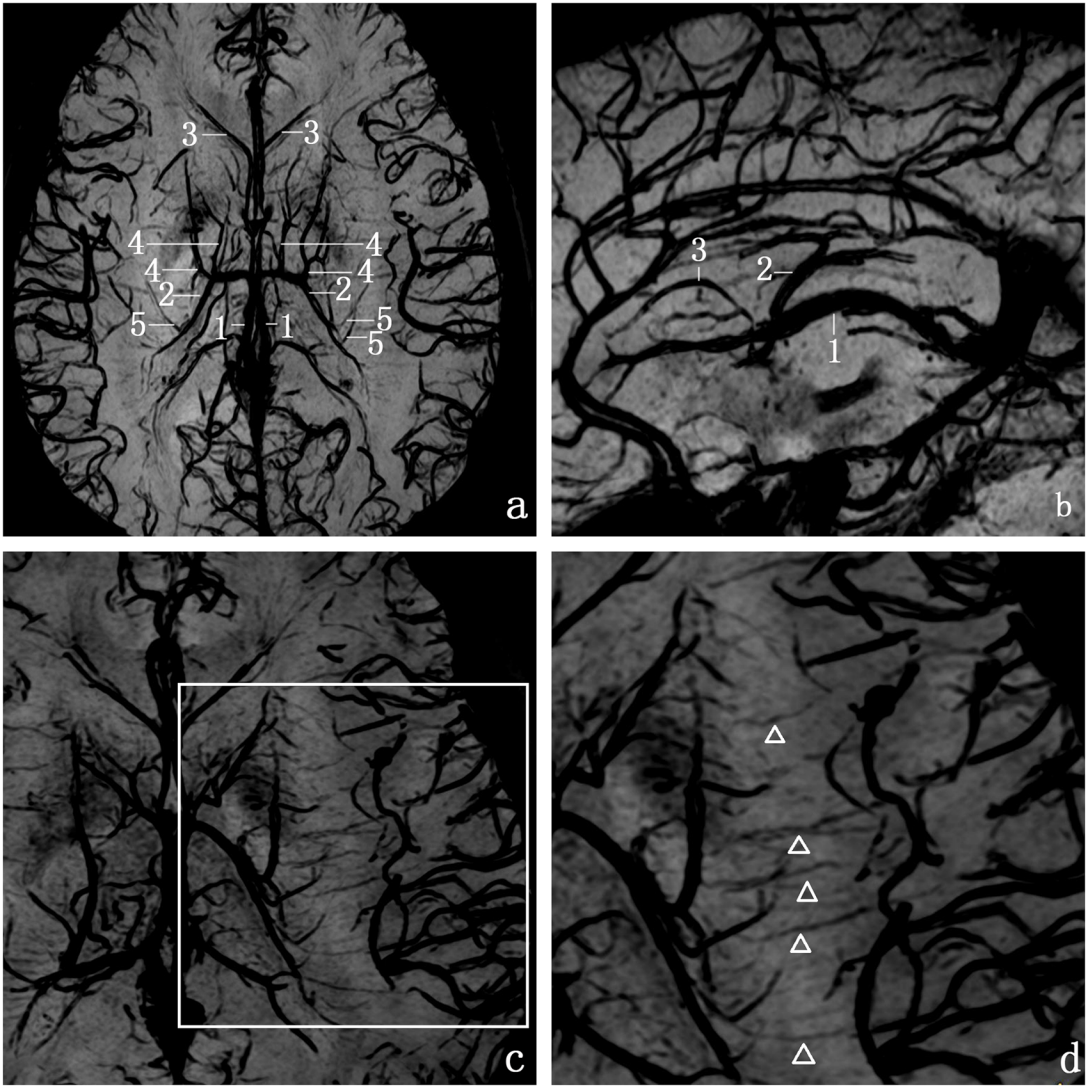
Transverse and sagittal SW images of the brain at 3.0 Tesla. a, transverse images; b, sigittal images; c and d, displaying the tiny deep medullary veins. (1,internal cerebral vein (ICV); 2,thalamostriate vein (TSV); 3, anterior septal vein (ASV); 4, anterior caudate vein (ACV); and 5,transverse caudate vein (TCV); the white arrowhead, small deep medullary veins).

Using mIP processing software and multiplanar reconstruction (MPR), SWI clearly showed TSV and its small tributaries (Fig 4a and 4b). The detection rates of each vein are summarized in Table 1. The TSV originates in the anterior wall of the atrium or in the inferolateral wall of the body of the lateral ventricle, and passing anteriorly and medially, beneath the stria terminalis it receives smaller tributaries on its way toward the foramen of Monro. The drainage areas of TSV include the caudate nucleus, internal capsule, lentiform nucleus, external capsule, claustrum, extreme capsule and the white matter of the frontoparietal lobes (Fig 4a and 4b). There were few veins of thalamus draining into TSV. Inthe transverse view, we also found small deep medullary veinswhich anastomose the tributaries of thalamostriate vein with the superficial medullary veins (Fig 4c and 4d).

**Table 1:** Frequencies of TSV and its tributaries.

Veins	Left, n (%)	Right, n (%)	Both sides, n (%)
Thalamostriate vein	39 (97.5)	35 (87.5)	74 (92.5)
Anterior caudate veins	35 (87.5)	35 (87.5)	70 (87.5)
Transverse caudate veins	27 (67.5)	24 (60)	51 (63.8)

### Variation of TSV

**Fig 5:**
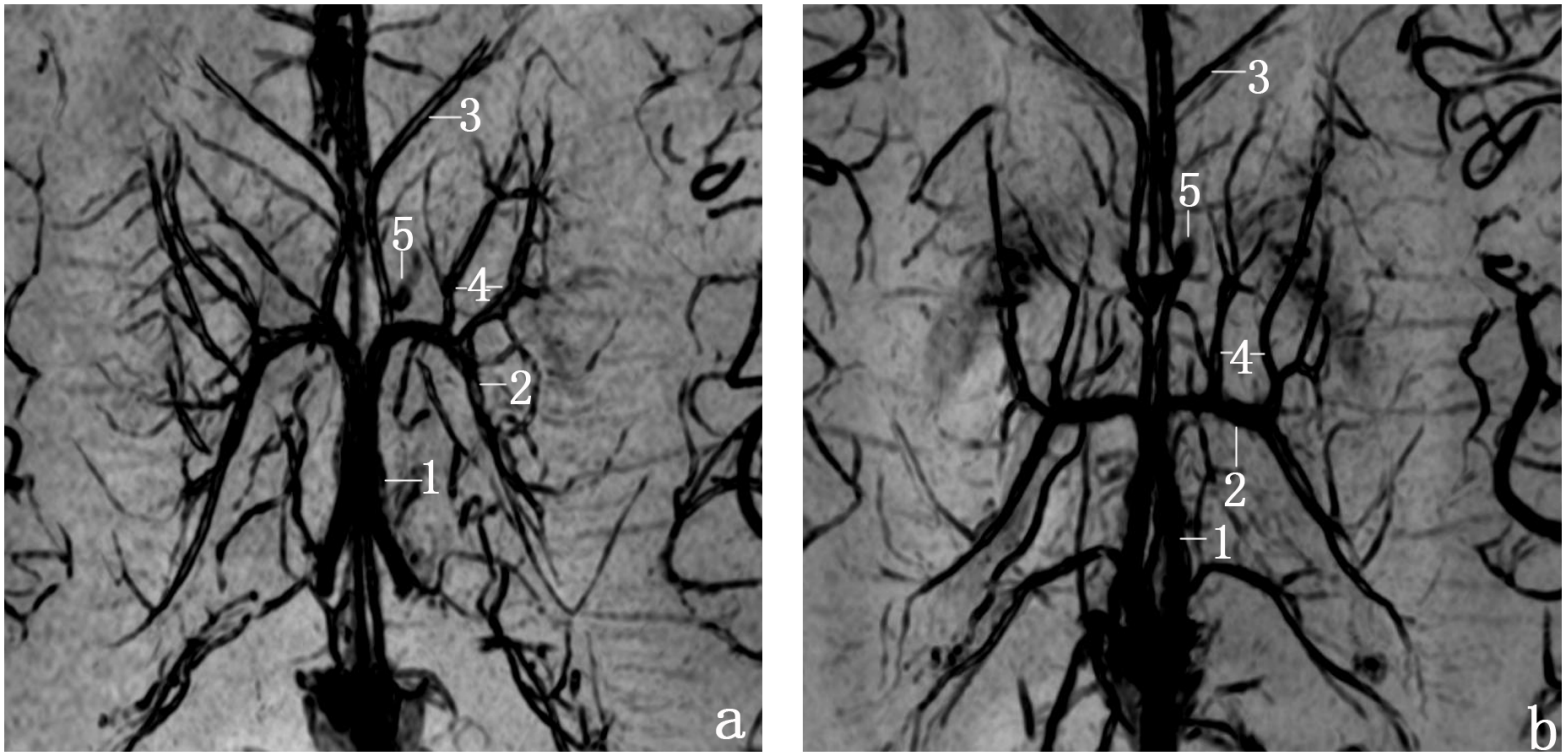
Anatomic variation of TSV types I and II on transverse SW images. a, Type Iforms venous angle; b, Type II forms false venous angle. (1, ICV; 2, TSV; 3, ASV; 4, ACV; and 5, the foramen of Monro).

Fig 5 shows representative cases of typesIand II. Table 2 summarizes the frequencies of the two types. In 59(79.7%) of 74 sides, the venous angle was formed (Fig 5a; Table 2). In 15(20.3%) of 74 sides, the false venous angle was formed (Fig 5b; Table 2). There were significant differences in the constitution ratios between the right and left hemispheres (*P* = 0.018 <0.05).

**Table 2:** Anatomic variation of TSV.

Types	Type of venous angle	Distance between FM and TSV(mm)	Number(n, %)			*P*
		Mean (range)	Both	Left	Right	
Type I	Venous angle	0 (0)	59 (79.7)	27 (69.2)	32 (91.4)	0.018
Type II	False venous angle	9.15 ± 4.09 (3.20–14.00)	15 (20.3)	12 (30.8)	3 (8.6)	

The distance between the foramen of Monro and TSV typeII was 9.15 ± 4.09mm.

### Variation of ACV

**Fig 6:**
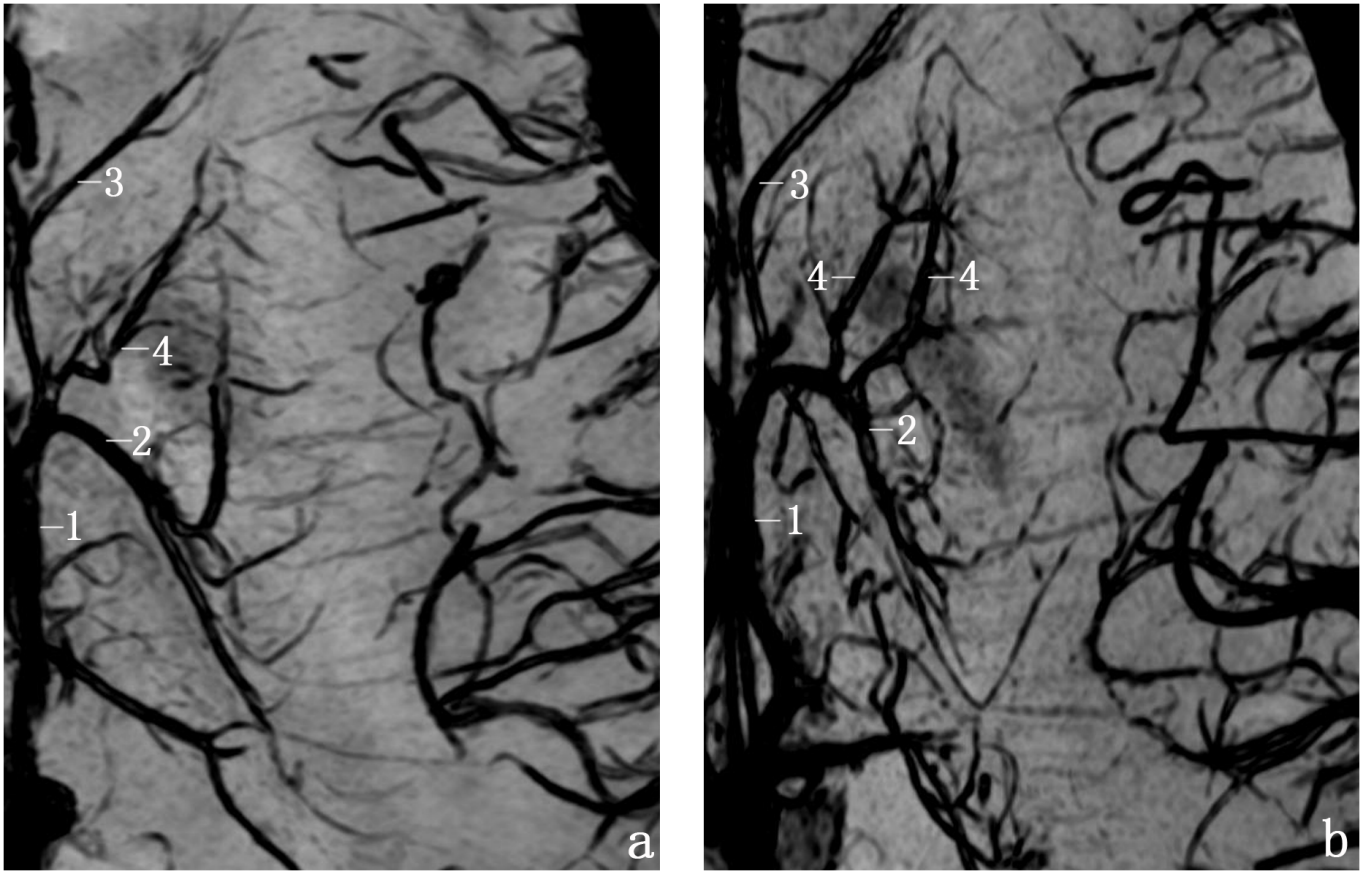
Transverse SWI of ACV types 1 and 2. a, Type 1 contains 1trunk; b, Type 2 contains 2 trunks. (1,ICV; 2,TSV; 3, ASV; and 4, ACV).

**Fig 7:**
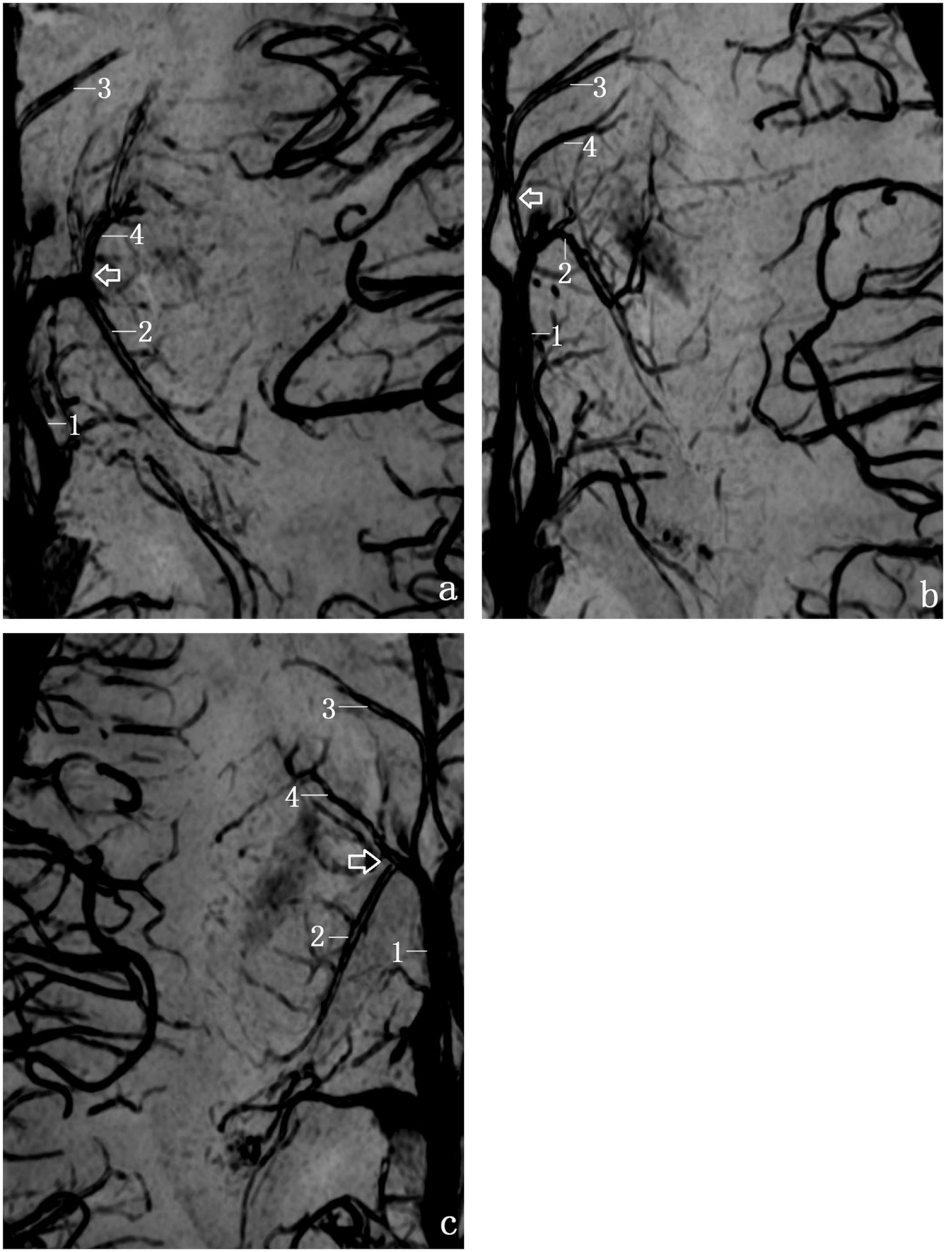
Transverse SWI of three different types of ACV(A, B and C). a, Type A (joining TSV as the white arrow indicates); b, Type B (joining the ASV as the white arrow indicates); c, Type C (joining the angle of TSV and ASV as the white arrow indicates). (1, ICV; 2, TSV; 3, ASV; 4, ACV).

Fig 6 clearly demonstrated the cases of types 1 and 2. In 63(90.0%) of 70 hemispheres, the ACV formed a single trunk (Fig 6a; Table 3). In 7 (10.0%) sides, the ACV finally formed 2 trunks (Fig 6b; Table 3). Fig 7 shows the typical cases of types A (64.3%), B (14.3%) and C (21.4%). There were no significant differences in ratios between the left and right sides (*P*>0.05)

**Table 3:** Anatomic variation of ACV.

Basis of classification	Types	Left, n (%)	Right, n (%)	Both, n (%)
Number of trunks	Type 1	30 (85.7)	33 (94.3)	63 (90.0)
	Type 2	5 (14.3)	2 (5.7)	7 (10.0)
Terminal joining position	Type A	25 (71.4)	20 (57.1)	45 (64.3)
	Type B	5 (14.3)	5 (14.3)	10 (14.3)
	Type C	5 (14.3)	10 (28.6)	15 (21.4)

## Discussion

SWI is a novel neuroimaging technique, based on differences in tissue magnetic susceptibility yielding a unique contrast, different from that of spin density, T1, T2, and T2*[15]. It displays small anatomic structures such as veins and deep nuclei with superb contrast and resolution, based on increased iron content, unlike conventional MRI. Compared with other imaging techniques, SWI does not require injection of contrast material. It is sensitive to veins, especially small veins. SWI is free from arterial contamination which is a major challenge with MRV. Rapid advances in medical imaging have resulted in the emergence ofultrahigh-field MRI systems of 7.0T [16–18]and 9.0 T [19]. SWI reveals deep cerebral venous anatomy [6, 20]. Itis a reliable tool to measure the cerebral venous diameter [5].

SWI is comparable to 3D CE-MRV in revealing TSV anatomy and provides better image resolution of ACVs [21]. In our study, the TSV and all the smaller tributaries were clearly visualized. We found the TSV, ACV and TCV in 92.5%, 87.5% and 63.8% of all hemispheres, respectively. Michio Ono [22]found the TSV and ACV in 90% and 100% of all autopsy samples, respectively. Compared with the anatomic study, the frequency of TSVwas high, but the ACV detection rate was low in our study, probably due to sample differences. The SW images showed that the TSV received small veins from the caudate nucleus, internal capsule, lentiform nucleus and the deep white matter of the frontoparietal lobes, but not from the thalamus, consistent with previousstudies [6, 23, 24]. Therefore, the TSV may be a misnomer. TSV might bebetter designated as frontoparietal internal vein,instead, as proposed byHassler [23].

In addition,we know that the abundance of TSV collateral circulation contributes to its safe occlusion in neurosurgery. Several deep medullary veins, which anastomose with the superficial medullary veins drain into the TSV. The small size of the anastomotic veins prevents detection with routine vascular imaging techniques. Juliane Budde [25]reported that these small veins were clearly demonstrated at 9.0T rather than 3.0 T. In our study, numerous small trans-medullary veins were clearly visible in the high-resolution images from 3.0 T (Fig 4d). The improved detection of tiny veins in our study may be related to better parameter setting and longer scanning time.

The junction of the TSV and internal cerebral vein (ICV)was seen adjacent to the posterior margin of the foramen of Monro called the venous angle or beyond it called the false venous angle. The foramen of Monro beyond the TSV-ICV junction can be enlarged to provide a wider access to the third ventricle without occluding the TSV. Several studies have described varying frequencies of the venous or false venous angle. Shinya Fujii [6]found the false venous angle in 19.1% using phase-sensitive imaging whereas N. Cagatay Cimsit [26] detected it in 34% of all subjects using MR time of flight(TOF) venography. The frequency of a false venous angle was 32.5% [14] or 39% [27] in two anatomical dissection studies. We observed the false venous angle in 20.3%. Therefore, the venous angle is the most common type suggesting possible damage to TSV during the neurosurgery of third ventricle. We also found a statistically significant difference in the ratios of venous angle in both sides (*P*<0.05), more commonly in the right hemispheres than the left. It suggests that abnormally enlarged TSV in the right side may pose a higher risk of obstruction of the foramen of Monro. Although few reports suggest vascular lesions as the cause of obstruction of the foramen of Monro, Jody Leonardo [28] reported a rare case report, which displayed an abnormally enlarged TSV, leading to a unilateral obstruction and right-sided hydrocephalus. The U junction of the false venous angle was found to be 4.3mm (3-7mm) beyond the posterior margin of the foramen of Monro in a previous anatomical study [14] whereas it was9.15 ± 4.09mm (3.20–14.00mm)in our study.

The ACVs originate in the ventricular aspect of the head of the caudate nucleus and always converge posteriorly to form 1 or 2 trunks to join the TSV stem [6]. In our study, most of the ACVs (90.0%) formed a single trunk and 64.3% of all ACVs joined the stem of TSV. The frequency of types B or C, which join the ASV or the angle between ASV and TSV was low compared with type A, without any significant differences in the ratios bilaterally.

## Conclusion

The venous architecture of TSV and its small tributaries is a complex three-dimensional (3D) network manifesting great variation. A thorough preoperative understanding of the variation offers significant practical neurosurgical guidance.

## References

[1] Rauscher A, Sedlacik J, Barth M, Haacke E m, Reichenbach J R. Nonnvasiveassessment of vascular architecture and function during modulated blood oxygenation using susceptibility weighted magnetic resonance imaging. Magn Reson Med.2005; 54(1):87–95. 15968657 10.1002/mrm.20520

[2] Reichenbach JR, Venkatesan R, Schillinger DJ, Kido DK, Haacke EM. Small vessels in the human brain: MR venography with deoxyhemoglobin as an intrinsic contrast agent. Radiology.1997; 204(1):272–277. 9205259 10.1148/radiology.204.1.9205259

[3] Waugh JR, Sacharias N. Arteriographic complications in the DSA era. Radiology.1992; 182(1):243–246. 1727290 10.1148/radiology.182.1.1727290

[4] Boeckh-Behrens T, Lutz J, Lummel N, Burke M, Wesemann T, Schopf V, et al Susceptibility-weighted angiography (SWAN) of cerebral veins and arteries compared to TOF-MRA. Eur J Radiol.2012; 81(6):1238–1245. 10.1016/j.ejrad.2011.02.057 21466929

[5] Xia XB, Tan CL. A quantitative study of magnetic susceptibility-weighted imaging of deep cerebral veins. J Neuroradiol.2013; 40(5):355–359. 10.1016/j.neurad.2013.03.005 23669499

[6] Fujii S, Kanasaki Y, Matsusue E, Kakite S, Kminou T, Oqawa T. Demonstration of cerebral venous variations in the region of the third ventricle on phase-sensitive imaging. AJNR Am J Neuroradiol.2010; 31(1):55–59. 10.3174/ajnr.A1752 19729543 PMC7964067

[7] Di Ieva A, Tschabitscher M, Galzio RJ, Grabner G, Kronnerwetter C, Widhalm G, et al The veins of the nucleus dentatus: anatomical and radiological findings. Neuroimage.2011; 54(1):74–79. 10.1016/j.neuroimage.2010.07.045 20659570

[8] Fujima N, Kudo K, Terae S, Ishizaka K, Yazu R, Zaitsu Y, et al Non-invasive measurement of oxygen saturation in the spinal vein using SWI: quantitative evaluation under conditions of physiological and caffeine load. Neuroimage.2011; 54(1):344–349. 10.1016/j.neuroimage.2010.08.020 20727413

[9] Ishizaka K, Kudo K, Fujima N, Zaitsu Y, Yazu R, Tha KK, et al Detection of normal spinal veins by using susceptibility-weighted imaging. J Magn Reson Imaging.2010; 31(1):32–38. 10.1002/jmri.21989 20027570

[10] Cossu M, Lubinu F, Orunesu G, Pau A, Sehrbundt Viale E, Sini MG, et al Subchoroidal approach to the third ventricle. Microsurgical anatomy.Surg Neurol.1984; 21(4):325–331. 6701763 10.1016/0090-3019(84)90109-5

[11] Timurkaynak E, Izci Y, Acar F. Transcavum septum pellucidum interforniceal approach for the colloid cyst of the third ventricle Operative nuance. Surg Neurol. 2006; 66(5):544–547. 17084209 10.1016/j.surneu.2006.03.033

[12] Hirsch JF, Zouaoui A, Renier D, Pierre-Kahn A. A new surgical approach to the third ventricle with interruption of the striothalamicvein. Acta Neurochir (Wien).1979; 47(3–4):135–147. 474208 10.1007/BF01406399

[13] Elhammady MS, Heros RC. Cerebral Veins: To Sacrifice or Not to Sacrifice, That Is the Question. World Neurosurg.2015; 83(3):320–324. 10.1016/j.wneu.2013.06.003 23791957

[14] Ture U, Yasargil MG, Al-Mefty O. The transcallosal-transforaminal approach to the third ventricle with regard to the venous variations in this region. J Neurosurg.1997; 87(5):706–715. 9347979 10.3171/jns.1997.87.5.0706

[15] Haacke EM, Mittal S, Wu Z, Neelavalli J, Cheng YC. Susceptibility-weighted imaging: technical aspects and clinical applications, part 1. AJNR Am J Neuroradiol.2009; 30(1):19–30. 10.3174/ajnr.A1400 19039041 PMC3805391

[16] Lupo JM, Banerjee S, Kelley D, Xu D, Vigneron DB, Majumdar S, et al Partially-parallel, susceptibility-weighted MR imaging of brain vasculature at 7 Tesla using sensitivity encoding and an autocalibrating parallel technique. Conf Proc IEEE Eng Med Biol Soc.2006; 1:747–750. 17945996 10.1109/IEMBS.2006.259807

[17] Rauscher A, Barth M, Herrmann KH, Witoszynskyi S, Deistung A, Reichenbach JR. Improved elimination of phase effects from background field inhomogeneities for susceptibility weighted imaging at high magnetic field strengths. Magn Reson Imaging.2008; 26(8):1145–1151. 10.1016/j.mri.2008.01.029 18524525

[18] Deistung A, Rauscher A, Sedlacik J, Stadler J, Witoszynskyi S, Reichenbach JR. Susceptibility weighted imaging at ultra high magnetic field strengths: theoretical considerations and experimental results. Magn Reson Med. 2008; 60(5):1155–1168. 10.1002/mrm.21754 18956467

[19] Budde J, Shajan G, Hoffmann J, Uqurbil K, Pohmann R. Human imaging at 9.4 T using T(2) *-, phase-, and susceptibility-weighted contrast. Magn Reson Med.2011; 65(2):544–550. 10.1002/mrm.22632 20872858

[20] Cai M, Zhang XF, Qiao HH, Lin ZX, Ren CG, Li JC, et al Susceptibility-weighted imaging of the venous networks around the brain stem. Neuroradiology. 2015; 57(2):163–169. 10.1007/s00234-014-1450-z 25326168

[21] Sun J, Wang J, Jie L, Wang H, Gong X. Visualization of the internal cerebral veins on MR phase-sensitive imaging: comparison with 3D gadolinium-enhanced MR venography and fast-spoiled gradient recalled imaging. AJNR Am J Neuroradiol. 2011; 32(10):E191–E193. 10.3174/ajnr.A2308 21163881 PMC7966027

[22] Ono M, Rhoton AJ, Peace D, Rodriquez RJ. Microsurgical anatomy of the deep venous system of the brain. Neurosurgery.1984; 15(5):621–657. 6504279 10.1227/00006123-198411000-00002

[23] Hassler O. Deep cerebral venous system in man. A microangiographic study on its areas of drainage and its anastomoses with the superficial cerebral veins.Neurology.1966; 16(5):505–511. 5949064 10.1212/wnl.16.5.505

[24] Wolf BS, Huang YP. THE SUBEPENDYMAL VEINS OF THE LATERAL VENTRICLES. Am J Roentgenol Radium Ther Nucl Med.1964; 91:406–426. 14118518

[25] Budde J, Shajan G, Hoffmann J, Uqurbil K, Pohmann R. Human imaging at 9.4 T using T(2) *-, phase-, and susceptibility-weighted contrast. Magn Reson Med.2011; 65(2):544–550. 10.1002/mrm.22632 20872858

[26] Cimsit NC, Ture U, Ekinci G, Necmettin Pamir M, Erzen C. Venous variations in the region of the third ventricle: the role of MR venography. Neuroradiology.2003; 45(12):900–904. 14551761 10.1007/s00234-003-1103-0

[27] Lang J. Surgical anatomy of the hypothalamus. Acta Neurochir (Wien).1985; 75(1–4):5–22. 3993452 10.1007/BF01406320

[28] Leonardo J, Grand W. Enlarged thalamostriate vein causing unilateral Monro foramen obstruction. Case report. J Neurosurg Pediatr.2009; 3(6):507–510. 10.3171/2009.2.PEDS0969 19485736

